# Simulation of action potential propagation based on the ghost structure method

**DOI:** 10.1038/s41598-019-47321-2

**Published:** 2019-07-29

**Authors:** Yongheng Wang, Li Cai, Xiaoyu Luo, Wenjun Ying, Hao Gao

**Affiliations:** 1 NPU-UoG International Cooperative Lab for Computation and Application in Cardiology, Northwestern Polytechnical University, Xi’an, 710129 China; 2 Xi’an Key Laboratory of Scientific Computation and Applied Statistics, Northwestern Polytechnical University, Xi’an, 710129 China; 3 School of Mathematics and Statistics, University of Glasgow, Glasgow, G12 8QQ UK; 4 Zhiyuan College, Shanghai Jiao Tong University, Shanghai, 200240 China

**Keywords:** Computational biology and bioinformatics, Mathematics and computing

## Abstract

In this paper, a ghost structure (GS) method is proposed to simulate the monodomain model in irregular computational domains using finite difference without regenerating body-fitted grids. In order to verify the validity of the GS method, it is first used to solve the Fitzhugh-Nagumo monodomain model in rectangular and circular regions at different states (the stationary and moving states). Then, the GS method is used to simulate the propagation of the action potential (AP) in transverse and longitudinal sections of a healthy human heart, and with left bundle branch block (LBBB). Finally, we analyze the AP and calcium concentration under healthy and LBBB conditions. Our numerical results show that the GS method can accurately simulate AP propagation with different computational domains either stationary or moving, and we also find that LBBB will cause the left ventricle to contract later than the right ventricle, which in turn affects synchronized contraction of the two ventricles.

## Introduction

The heart is a rhythmic pump that maintains blood circulation throughout the body^1^. The rhythmic beating of the heart is caused by the regular spread of action potential (AP) within the heart. Abnormal conduction of AP in the heart can cause arrhythmias. Symptoms of arrhythmia include extrasystole, tachycardia, ventricular fibrillation, etc., of which ventricular fibrillation is the leading cause of cardiac sudden death^2,3^. Sudden cardiac death accounts for 15% of global deaths, and about 80% of sudden cardiac death is the result of ventricular arrhythmias^4^. Furthermore, about a quarter of patients with heart failure are diagnosed with LBBB^5^, which causes asynchronous AP propagation and contraction of the left ventricle, and then potentially leads to the global left ventricle dysfunction^6^. Therefore, it is of great significance to study the mechanism of arrhythmia, such as through numerical modelling, which can explore extreme situations that is difficult to perform in experiments. For several decades, the electrical activity of the heart has been modeled by a system of singularly perturbed reaction-diffusion partial differential equations that couples a set of ordinary differential equations used to describe the cell membrane dynamics^7,8^. The effects of different types of the electrical stimulation on arrhythmia can then be studied by solving these differential equations numerically. At present, numerical simulation of electrical activity has become a powerful tool for studying and understanding cardiac electrophysiology and arrhythmia^9–11^.

To mathematically model cardiac action potential, a cardiomyocyte model is required. With the abundance of experimental data, myocyte models have been continuously improved. A large number of mammalian cardiomyocyte models already exist in the literature^12^, such as Beeler-Reuter model^13^, Luo-Rudy model^14^, Fenton-Karma model^15^, etc. In order to accurately study the human heart, a large number of human cardiomyocyte models have also been proposed, such as ten Tusscher model^16^, Grandi-Pasqualini-Bers (GPB) model^17^, etc. For example, the GPB model can be used to describe Ca^2+^ handling and ionic currents in human ventricular myocytes, and its effectiveness has been validated against available experimental data^7,17,18^. In 1969, Schmitt *et al*.^8^ proposed a bidomain model for AP propagation in tissue level, then was further developed in the late 1970s^19–21^. The bidomain model describes active cardiomyocytes on a macroscopic scale by membrane ion current, membrane potential and extracellular potential^22^. Based on a given membrane potential, the bidomain model can modelling both the extracellular potential and the body-surface potential^23^. Recently, Bendahmane *et al*.^24^ introduced a “stochastically forced” version of the bidomain model that accounted for various random effects, and further established the existence of weak solutions to the stochastic bidomain model, which was proved by means of an auxiliary nondegenerate system and the Faedo-Galerkin method. The bidomain model is considered to be the most complete model to describe the electrical activity of the heart^25,26^. However, solving the bidomain equations is computationally expensive because of the required fine spatial and temporal discretization, which limits the size and duration of the problem that can be modeled^27^. The monodomain model is a simplification of the bidomain model. Compared with the bidomain model, the monodomain model is less computational demanding^28,29^, has been widely used to simulate AP propagation^30,31^. Cloherty *et al*.^32^ developed a biophysically detailed two-dimensional monodomain model of the rabbit sinoatrial node and surrounding atrial tissue. This model yielded new insights into the mechanisms of AP propagation from the sinus node to the atrium, such as the effects of vagal stimulation on pacemaker position. Belhamadia *et al*.^33^ simulated the electrophysiological waves of a three-dimensional heart through a monodomain simulation by proposing an accurate numerical method based on a time-dependent anisotropic remeshing strategy, which greatly reduced the number of elements and enhanced the accuracy of the prediction of the electrical wave fronts. Kunisch *et al*.^34^ proposed an optimal control approach to a simplified reaction-diffusion system describing cardiac defibrillation, which allowed for joint optimization of shape and duration of defibrillation pulses. Various cell membrane models have been used with the monodomain model, but some models are not based on any experimentally measured quantities, such as the the simplest and most widely used FitzHugh-Nagumo model^35^. The Ftzhugh-Nagumo monodomain model has been used to describe the propagation of potential in heterogeneous heart tissues^36,37^.

Different numerical methods have been developed to solve the monodomain model. Zhang *et al*.^38^ used the element-free Galerkin method for studying the effects of myocardial geometrical complexity, material inhomogeneity, and material anisotropicness on the electrical transmission. Shuaiby *et al*.^36^ presented a finite element method which coupled with the modified FitzHugh-Nagumo model in the simulation of the cardiac excitation isotropic propagation. Rahman *et al*.^37^ used the Galerkin finite element method to solve the FitzHugh-Nagumo monodomain model. Cai *et al*.^39^ proposed a completely discrete implicit-explicit finite element scheme for solving the FitzHugh-Nagumo monodomain model. In this scheme, a simple linearization technique used to make the process of solving equations more efficient. The numerical results were reported to verify the convergence results and the stability of the scheme. Liu *et al*.^40^ proposed a fractional Fitzhugh-Nagumo monodomain model with zero Dirichlet boundary conditions. Later, Liu^41^ further developed a decoupling technique to solve the fractional FitzHugh-Nagumo monodomain model, and proposed a new spatially second-order accurate semi-implicit alternating direction method to solve this model on approximate irregular regions.The model generalized a standard monodomain model that described the propagation of potentials in heterogeneous cardiac tissue. Bu *et al*.^42^ developed a new Crank-Nicolson alternating direction implicit Galerkin finite element method and discussed the stability and convergence of method.

Modelling LBBB will continue to deepen our understanding on its pathology and treatments, including the mechanical discoordination^43^, electrical dyssynchrony^44^ and their interactions^45^. Kevin *et al*.^46^ mapped the left ventricle endocardial electrical activation, myocardial circumferential shortening, and myocardial blood flow of LBBB at different time, and then employed a serial two-dimensional echocardiography to assess the ventricular remodelling. The results showed that asynchronous ventricular activation would affect myocardial circumferential shortening and myocardial blood flow, and eventually lead to the left ventricle remodelling. Lange *et al*.^44^ proposed a computational model of human heart that included a false tendons, Purkinje network, and papillary muscles, and investigated effects of different types of false tendons on hearts with electrical conduction abnormality caused by LBBB. They found that the false tendons could be visualized as an alternative conduction pathway, and compensates for propagation delay with LBBB. Kerckhoffs *et al*.^43^ employed a computational model of a LBBB heart to model asymmetric hypertrophy, their results showed that LBBB led to a step increase in left ventricle mechanical discoordination. Usyk *et al*.^47^ developed a three-dimensional model of a dilated failing heart with LBBB, and investigated how biventricular pacing could improve systolic mechanical performance and synchrony.

The heart has a complex geometry. When a general difference scheme is used to solve the heart potential propagation, finite difference discretization of higher-order derivatives at irregular boundaries can be very complicated and challenging. To address this, we propose a ghost-structure (GS) method in this study, in which the transmembrane potential is described by the Eulerian form, while the membrane dynamics, including ion concentration, stimulation current density, and ion current, are described by the Lagrangian form. The transformation between the Lagrangian variable and the Eulerian variable is achieved by an integral transformation with a delta function. This GS method can solve the monodomain model in the moving region which is similar to that of immersed boundary method^18,48–51,52^ in dealing with fluid-solid coupling problems.

The remaining of the paper is organised as follows. The section “Results” verifies the validity of the GS method through various numerical examples. In the verification example, we simulate the two-dimensional Fitzhugh-Nagumo monodomain model on a rectangular and circular regions. For AP propagation in human ventricles, we compare AP propagations in a healthy heart and a disease heart with LBBB. Then followed by “Discussion” and “Conclusion”. In the section “Model introduction”, we discuss the bidomain and monodomain model, and reaction-diffusion systems. In the section “The ghost structure method”, we introduce the ghost structure method and its discrete scheme in space and time.

## Results

### FitzHugh-Nagumo monodomain model

In this section, some numerical examples are given to verify the efficiency of the proposed GS method. In neurons, numerous types of ion channels can influence the membrane potential. The voltage-gated ion channels are controlled by the membrane potential, while the membrane potential is influenced by these same ion channels, which causes feedback loops which allow for complex temporal dynamics, including oscillations and regenerative events such as AP. Firstly, the GS method is used to solve a two-dimensional FitzHugh-Nagumo model in a rectangular and circular regions^40,53^. The FitzHugh-Nagumo model is

1 $$\frac{\partial u}{\partial t}={K}_{x}\frac{{\partial }^{2}u}{\partial {x}^{2}}+{K}_{y}\frac{{\partial }^{2}u}{\partial {y}^{2}}+{I}_{ion}$$

2 $${I}_{ion}=u(1-u)(u-a)-v$$

3 $$\frac{\partial v}{\partial t}=\varepsilon (\beta u-\gamma v-\sigma )$$

where, *u* is a normalized transmembrane potential, *v* is a recovery variable. *K*_*x*_ and *K*_*x*_ are the components of diffusion coefficient **K**. The model parameters *a* = 0.1, *ε* = 0.01, *β* = 0.5, *γ* = 1, *σ* = 0. The rectangular region is [0, 2.5] × [0, 2.5] with zero Dirichlet boundary conditions, and the initial conditions in this rectangular case are chosen as

4 $$u(x,y,\mathrm{0)}=\{\begin{array}{ll}\mathrm{1.0,} & 0 < x\le 1.25,0 < y < 1.25\\ \mathrm{0.0,} & 1.25\le x < 2.5,0 < y < 1.25\\ \mathrm{0.0,} & 0 < x\le 1.25,1.25\le y < 2.5\\ \mathrm{0.0,} & 1.25\le x < 2.5,1.25\le y < 2.5\end{array}$$

5 $$v(x,y,\,0)=\{\begin{array}{ll}0.0, & 0 < x\le 1.25,0 < y < 1.25\\ 0.0, & 1.25\le x < 2.5,0 < y < 1.25\\ 0.1, & 0 < x\le 1.25,1.25\le y < 2.5\\ 0.1, & 1.25\le x < 2.5,1.25\le y < 2.5\end{array}$$

Relevant numerical results of this example can be found in the literature^40,53^, including stable spiral waves. In order to validate the GS method, the computational domain of the regular ghost structure is taken as [−0.1, 2.6] × [−0.1, 2.6] with 275 × 275 grids with a time step of *dt* = 0.1.

**Figure 1:**
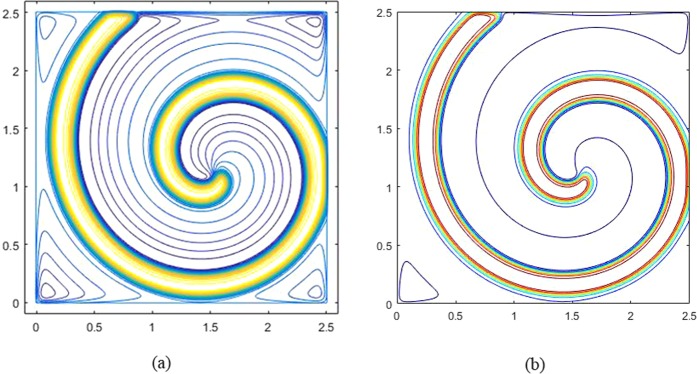
Spiral waves in the Fitzhugh-Nagumo model at t = 1000: (**a**) result of GS method; (**b**) result of Liu.

In this Fitzhugh-Nagumo model, when *K*_*x*_ = *K*_*y*_ = 10^−4^, Fig. 1 gives the spiral wave of the stable rotation solution at *t* = 1000. As can be seen from Fig. 1, for the rectangular region, the spiral wave of the model generates a clockwise rotation curve. Figures 1(a,b) give the spiral waves obtained by the GS method and Liu^40,53^, respectively. It can be found that the spiral wave structure obtained by the GS method is consistent with the results obtained by Liu^40^.

**Figure 2:**
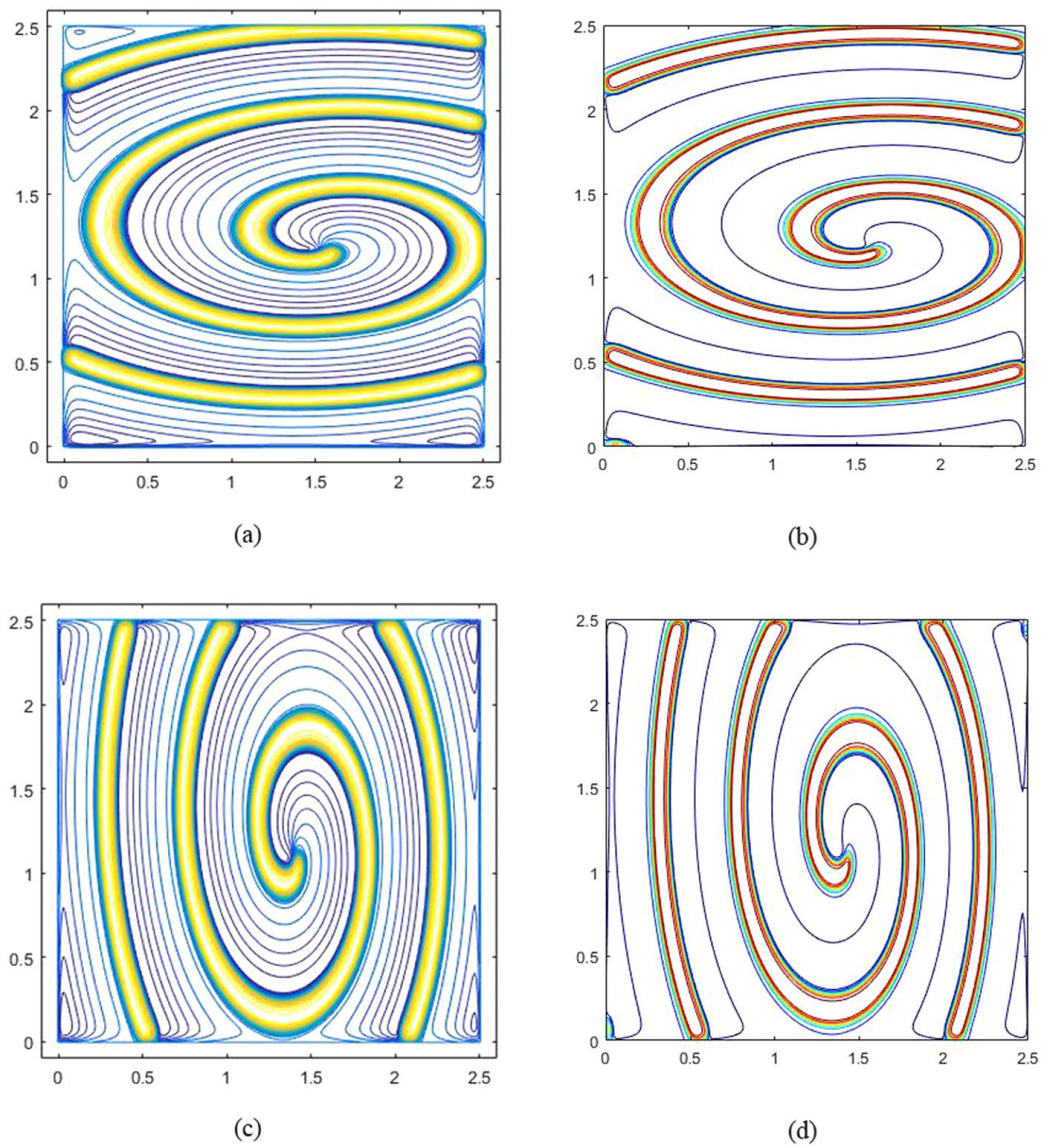
Spiral waves in the Fitzhugh-Nagumo model with anisotropic diffusion ratios at t = 1000, (**a**) *K*_*x*_ = 10^−4^, *K*_*y*_ = 0.25 × *K*_*x*_, result of the GS method; (**b**) *K*_*x*_ = 10^−4^, *K*_*y*_ = 0.25 × *K*_*x*_, result of Liu; (**c**) *K*_*y*_ = 10^−4^, *K*_*x*_ = 0.25 × *K*_*y*_, result of the GS method; (**d**) *K*_*y*_ = 10^−4^, *K*_*x*_ = 0.25 × *K*_*y*_, result of Liu.

Figure 2(a,b) show the numerical results obtained by the GS method and Liu *et al*.^40,53^ with *K*_*x*_ = 10^−4^, *K*_*y*_/*K*_*x*_ = 0.25, and Fig. 2(c,d) are the results with *K*_*y*_ = 10^−4^, *K*_*x*_/*K*_*y*_ = 0.25. It can be found that for *K*_*x*_ = 10^−4^, *K*_*y*_/*K*_*x*_ = 0.25 and *K*_*y*_ = 10^−4^, *K*_*x*_/*K*_*y*_ = 0.25, the spiral wave structures obtained by the GS method are nearly identical as those obtained by Liu *et al*.^40,53^.

Secondly, using the same model parameters and boundary condition, the computational domain is changed to be a circle Ω = {(*x*, *y*)|(*x* − 1.25)^2^ + (*y* − 1.25)^2^ ≤ 1.25^2^} with the following initial conditions:

6 $$u(x,y,\,0)=\{\begin{array}{ll}\mathrm{1.0,} & 1.25-\sqrt{{1.25}^{2}-{\mathrm{(1.25}-y)}^{2}} < x\le \mathrm{1.25,\ 1.25}-\sqrt{{1.25}^{2}-{\mathrm{(1.25}-x)}^{2}}\\  &  < \,y\le 1.25\\ \mathrm{0,} & other\end{array}$$

7 $$v(x,y,\,0)=\{\begin{array}{ll}0.1, & 1.25-\sqrt{{1.25}^{2}-{\mathrm{(1.25}-y)}^{2}} < x < 1.25+\sqrt{{1.25}^{2}-{\mathrm{(1.25}-y)}^{2}},\\  & \,1.25\le y < 1.25+\sqrt{{1.25}^{2}-{\mathrm{(1.25}-x)}^{2}}\\ 0, & other\end{array}$$

**Figure 3:**
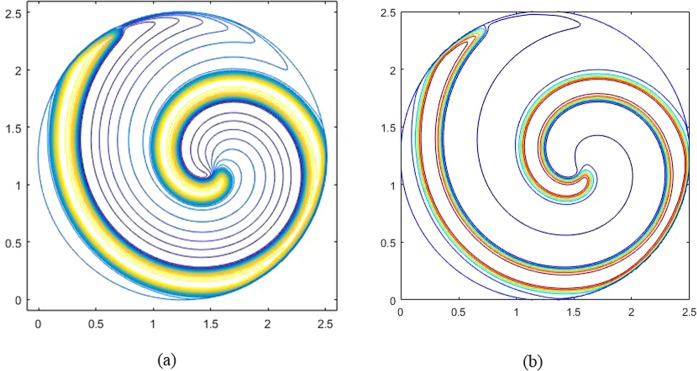
Spiral waves in the Fitzhugh-Nagumo model at t = 1000: (**a**) result of the GS method; (**b**) result of Liu.

The computational domain of the ghost structure remains the same as the rectangular case. Figure 3(a,b) show the numerical results obtained by the GS method and Liu^41^ with *K*_*x*_ = *K*_*y*_ = 10^−4^ at t = 1000. It can be seen from Fig. 3 that the results obtained by the GS method in the circular region are also identical to those obtained by Liu^41^.

**Figure 4:**
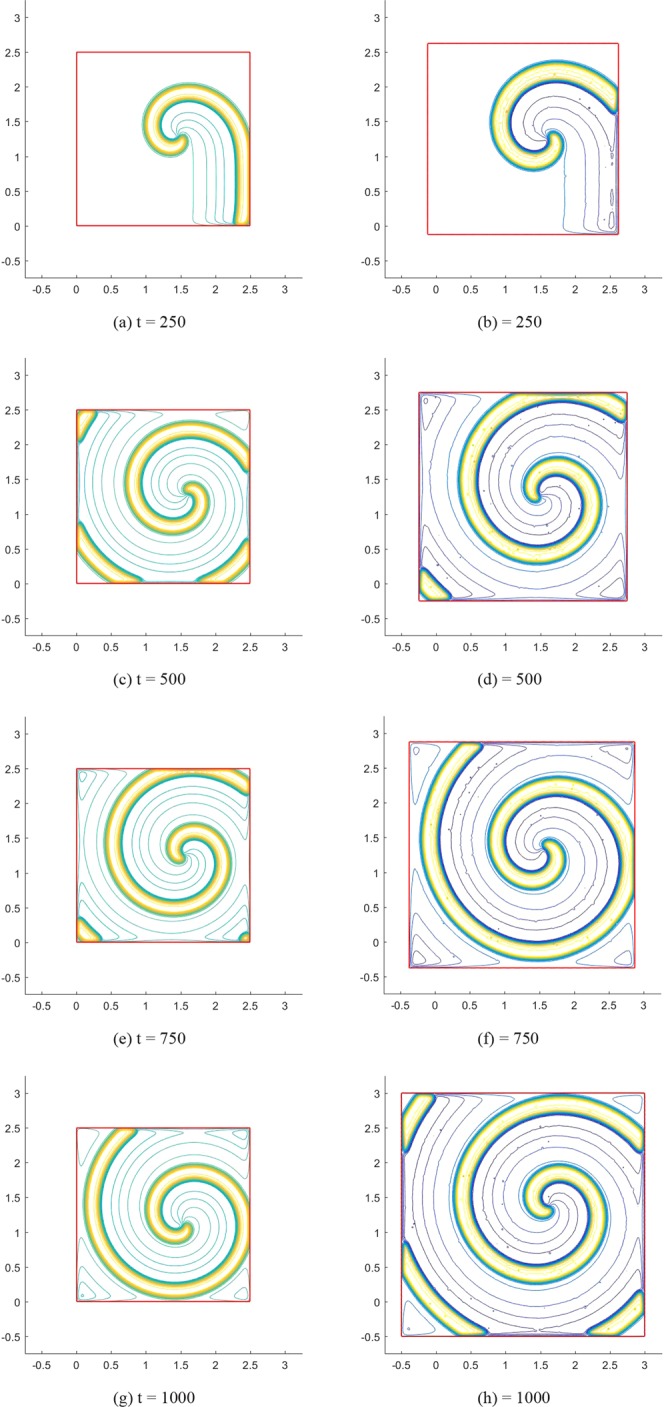
Spiral waves in the Fitzhugh-Nagumo model: the left figures show the results in a stationary rectangular region; the right figures show the results in a moving rectangular region.

**Figure 5:**
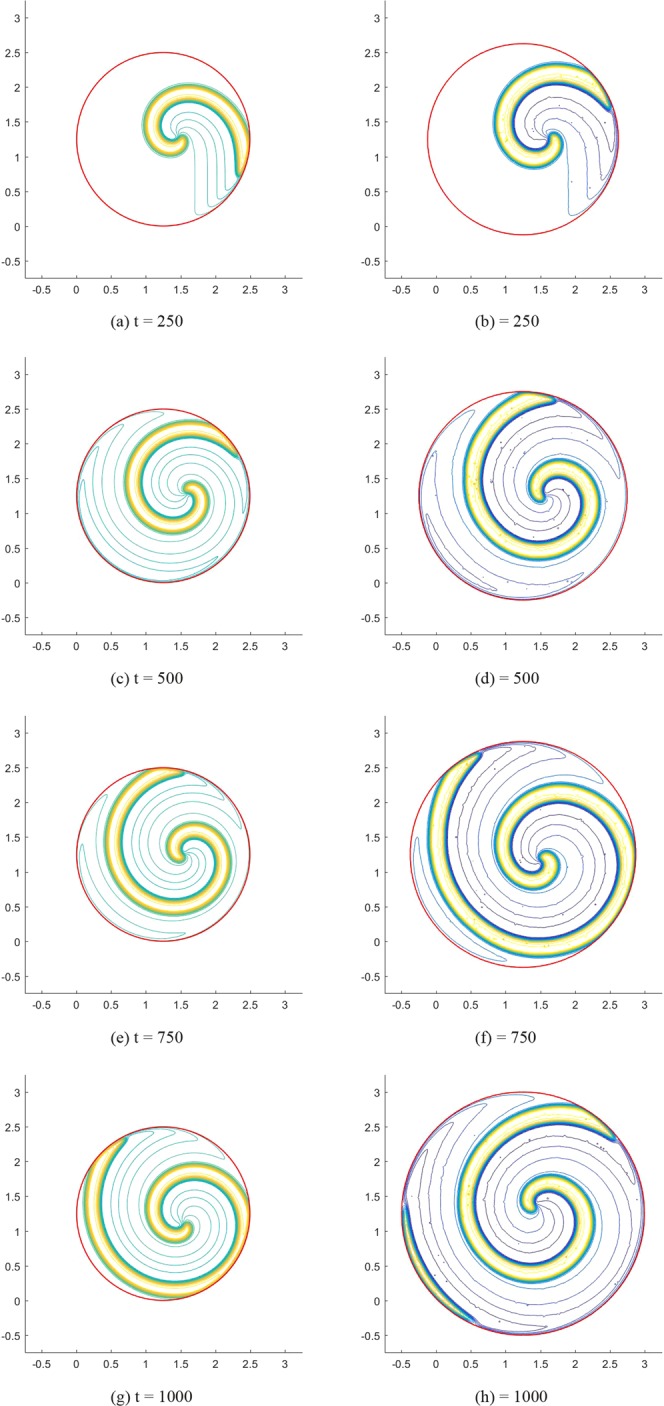
Spiral waves in the Fitzhugh-Nagumo model: the left figures show the results in a stationary circular region; the right figures show the results in a moving circular region.

Finally, we simulate the transmembrane potential propagation in moving regions by using the same model parameters and boundary condition. To do this, we assume the Lagrangian point in the rectangular and circular region mentioned above expands in the normal direction $\overrightarrow{n}({\bf{X}})=\frac{{\bf{X}}-{{\bf{X}}}_{c}}{{\Vert {\bf{X}}-{{\bf{X}}}_{c}\Vert }_{2}}$ with a velocity $k({\bf{X}})=\frac{0.5{\Vert {\bf{X}}-{{\bf{X}}}_{c}\Vert }_{2}}{1.25}$, where **X**_*c*_ describes the centroid coordinate of the computational domain. The physical position of each Lagrangian point at time t, namely *χ*(**X**, *t*), satisfies $\frac{\partial \chi ({\bf{X}},t)}{\partial t}=k({\bf{X}})\overrightarrow{n}({\bf{X}})$. The computational domain of the regular ghost structure is taken as [−0.75, 3.25] × [−0.75, 3.25] with 408 × 408 grids, and the time step *dt* = 0.1, which is the same as the time step of potential propagation. For *K*_*x*_ = *K*_*y*_ = 10^−4^, the right figures in the Figs 4 and 5 give the transmembrane potential propagation in the moving rectangular and circular regions at different times, respectively. The left figures are from corresponding stationary regions. By comparing Figs 4 with 5, it can be found that the moving region affects both the propagation velocity and shape of spiral waves. The mean propagation velocities at point (1.5, 1.5) in the moving rectangular and circular regions are 0.134 and 0.137, respectively, which are higher than the values in the stationary regions (0.116 and 0.115). The width of the spiral wave in moving regions (rectangular region: 0.304, circular region: 0.306) is also slightly larger than the width in stationary region (rectangular region: 0.273, circular region: 0.271).

### AP propagation on ventricular section

**Figure 6:**
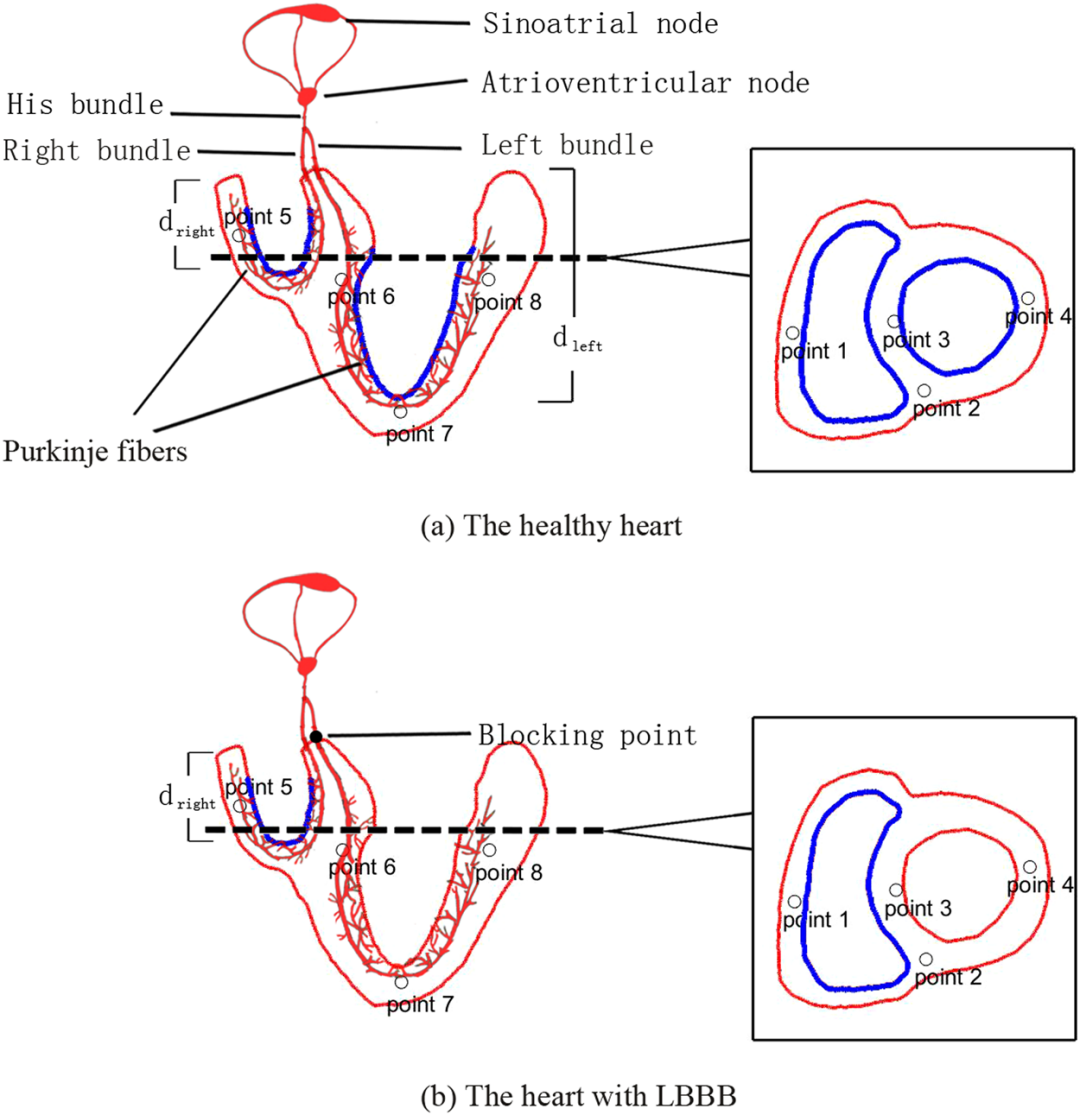
Sketch for electrical conduction system and the sections of human heart: the left figure shows the longitudinal section, and the right figure shows the transverse section of heart at the dotted line position.

The electrical conduction system of the heart triggers myocardial contraction by electrical impulses transmitted through the sinus node. As shown in Fig. 6, electrical pulses pass through the atrium to the atrioventricular node and enter the ventricle along the left bundle branch, right bundle branch and Purkinje fiber. When an electrical pulse is transmitted to the cardiomyocytes and triggers the AP production, an excitation-contraction coupling occurs in cardiomyocytes. Myocardial contraction is highly dependent on the dynamics of calcium in a single myocardial cell^54^, which is becasue myofilament contraction is regulated by an increase of the intracellular calcium transient (CaT). Therefore, the excitation-contraction coupling of myocytes essentially depends on the calcium-induced calcium release^55^.

In this section, we employ the GPB model to model the myocyte electrophysiology, which describes various ionic current *I*_*ion*_ and Ca^2+^ dynamics. The reasons for choosing the GPB model are that (1) it matches experimental data well^17^; (2) it is adequate to analyse AP with detailed Ca^2+^ dynamics, which plays a crucial role in excitation-contraction in myocardium^54^. In order to understand the AP propagation in ventricles, we select one transverse and one longitudinal sections obtained from a real human heart^51,56,57^. The computational domain for the ghost structure of the transverse and longitudinal sections of the ventricle are 130 *mm* × 110 *mm* and 140 *mm* × 140 *mm*, respectively, and the spatial step size in each direction is 0.14 *mm*. The discrete time step is 0.08 *ms*, the membrane capacitance *C*_*m*_ = 1 *μF*/*cm*^2^ and the surface-to-volume ratio^58^ *Am* = 0.24 *μm*^−1^. The human ventricular conductivity is listed in Table 1. ${\sigma }_{i}^{L}$ and ${\sigma }_{e}^{L}$ are the longitudinal intra- and extracellular conductivities, respectively. ${\sigma }_{i}^{t}$ and ${\sigma }_{e}^{t}$ are the transversal intra- and extracellular conductivities, respectively. As shown in Table 1, the ventricular conductivity depends on the region and direction of cardiomyocyte. In this study, we employ the data obtained by Potse *et al*.^28^ in Table 1.

**Table 1:** Conductivity values of human ventricle.

	Clerc^73^ (1976)	Roberts and Scher^74^ (1982)	Colli-Franzone^75^ (1993)	Potse^28^ (2006)
${\sigma }_{i}^{L}$	0.174	0.344	0.3	0.3
${\sigma }_{i}^{t}$	0.0193	0.0596	0.0315	0.03
${\sigma }_{e}^{L}$	0.625	0.117	0.2	0.3
${\sigma }_{e}^{t}$	0.236	0.0802	0.1341	0.12

The right and left bundle branches and Purkinje are the main conduction system in the ventricles. The right and left boundle branches divide into a few major branches and subsequently into Purkinje fibers^59^ as shown in Fig. 6. Purkinje fibers penetrate into the ventricular muscle, entangled in endocardium, and form a network. The network of Purkinje fibers do not contribute to the activation of ventricular muscles until it reaches the middle and lower third of the septum and ventricle^45^. Purkinje fibers allow for rapid, coordinated, and synchronous physiologic depolarization of the ventricles. Therefore, we consider the initial electrical stimulation to be located in the middle and lower third segments of the septum and endocardium^60^, as indicated by the blue region in Fig. 6(a). For a healthy heart in Fig. 6(a), the blue region of endocardial surface will have the electrical stimulus transmitted from the Purkinje network. The LBBB refers to the blockage of the left bundle branch conduction. In this study, we consider complete left bundle branch block. Thus, no electrical stimulus is transmitted in the left branch, but through the interventricular septum from the right ventricular endocardium to the left ventricle endocardium^45,61^. As shown in the Fig. 6(b), only the blue region in the right ventricle receives the electrical stimulus from the Purkinje fibers.

**Figure 7:**
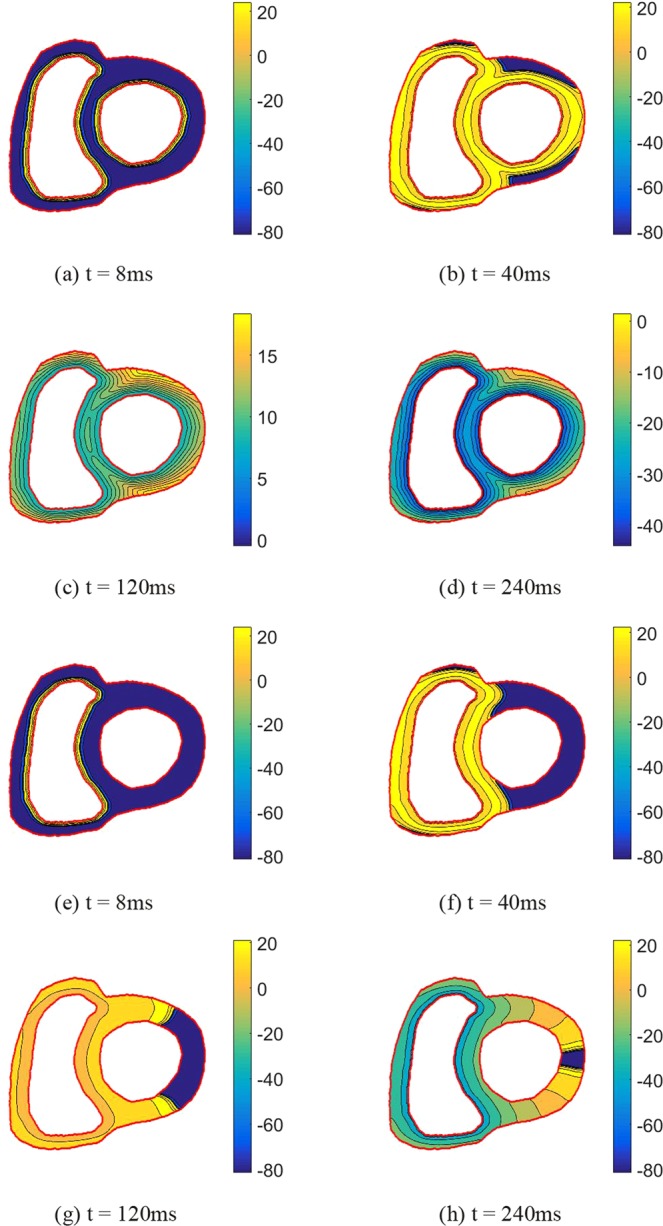
The AP propagation of transverse section of human heart at different times: The first row (**a**–**d**) illustrates the AP propagation of the healthy heart; The second row (**e**–**h**) illustrates the AP propagation of the heart with LBBB.

**Figure 8:**
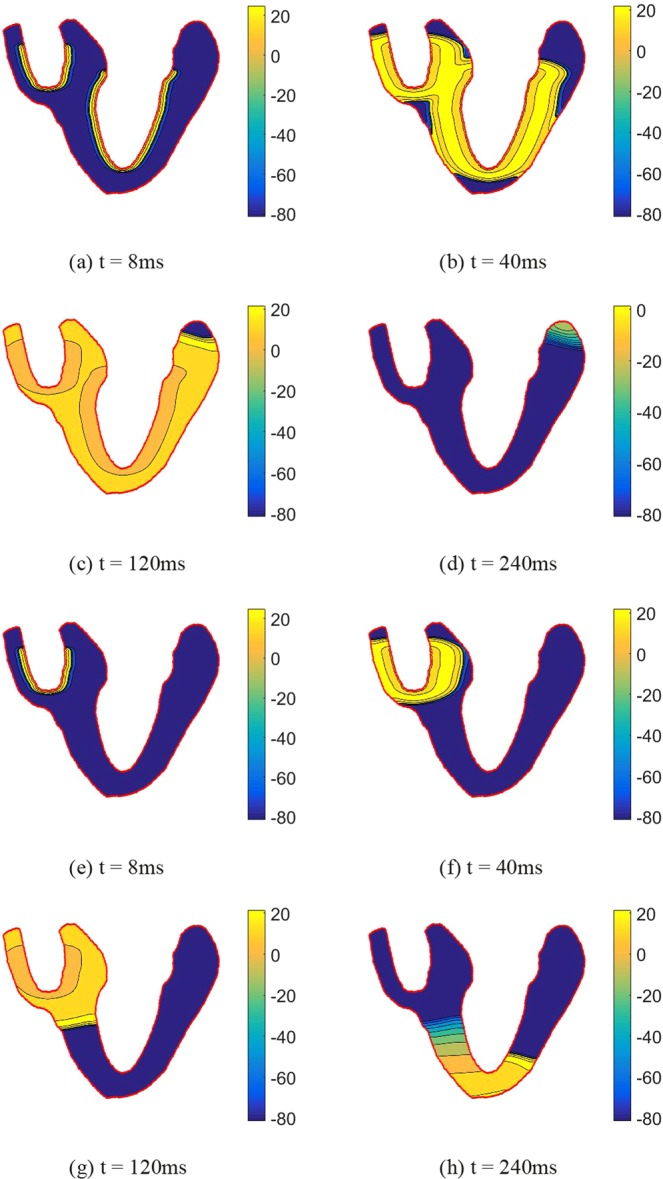
The AP propagation of longitudinal section of human heart at different times: (**a–d**) illustrates the AP propagation of the healthy heart; (**e–h**) illustrates the AP propagation of the heart with LBBB.

To understand the effects of LBBB, we now compare AP propagations in a healthy heart with and without LBBB. Figure 7 shows AP propagation in the transverse section of heart at different times. Figure 7(a–d) illustrate the state of AP propagation under healthy condition. Figure 7(e–h) shows AP propagation across the transverse section of heart with LBBB. Figure 8 shows AP propagation on the longitudinal section of heart. In the transverse section of heart, the healthy heart completes the AP propagation within approximately 347.2 *ms*, which is faster than the propagation with LBBB (540 *ms*). As shown in Fig. 7, under healthy conditions, when *t* = 40 *ms*, the AP spreads to most of the right ventricle and half of the left ventricular region. However, in the LBBB case, only half of the right ventricular region is excited, and the AP has not yet arrived at the left ventricular. When *t* = 120 ms, the whole healthy heart is repolarized, but still half of the left ventricle is not excited in the LBBB case. In the longitudinal section, the heart with LBBB needs 1011.2 ms to complete the AP propagation, which is 2.4 times the time (416 *ms*) that the healthy heart completes the propagation. As shown in Fig. 8, at *t* = 120 *ms*, almost the whole healthy heart is stimulated, but for the heart with LBBB, only the right ventricle is stimulated. When *t* = 360 *ms*, the AP only spreads to the apex of the heart with LBBB, while in the healthy heart, almost all regions return to the resting potential. Therefore, the LBBB causes significant delay in the activation of the left ventricle.

**Figure 9:**
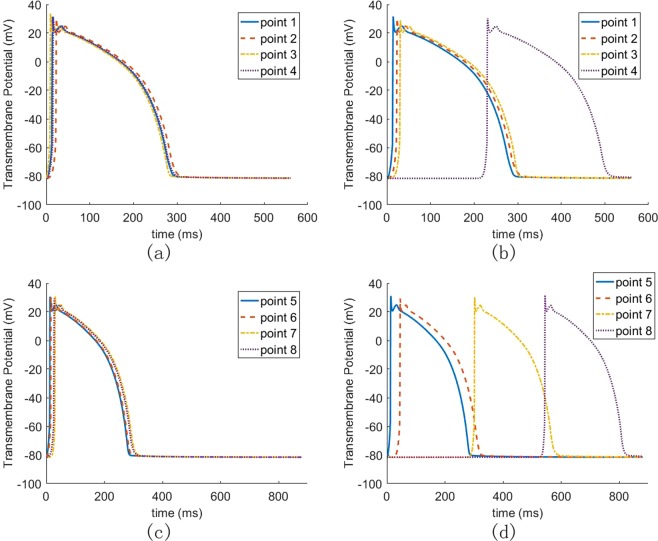
The AP propagation of human heart in different points: (**a**) four points in transverse section of the healthy heart; (**b**) four points in transverse section of the heart with LBBB; (**c**) four points in longitudinal section of the healthy heart; (**d**) four points in longitudinal section of the heart with LBBB.

**Figure 10:**
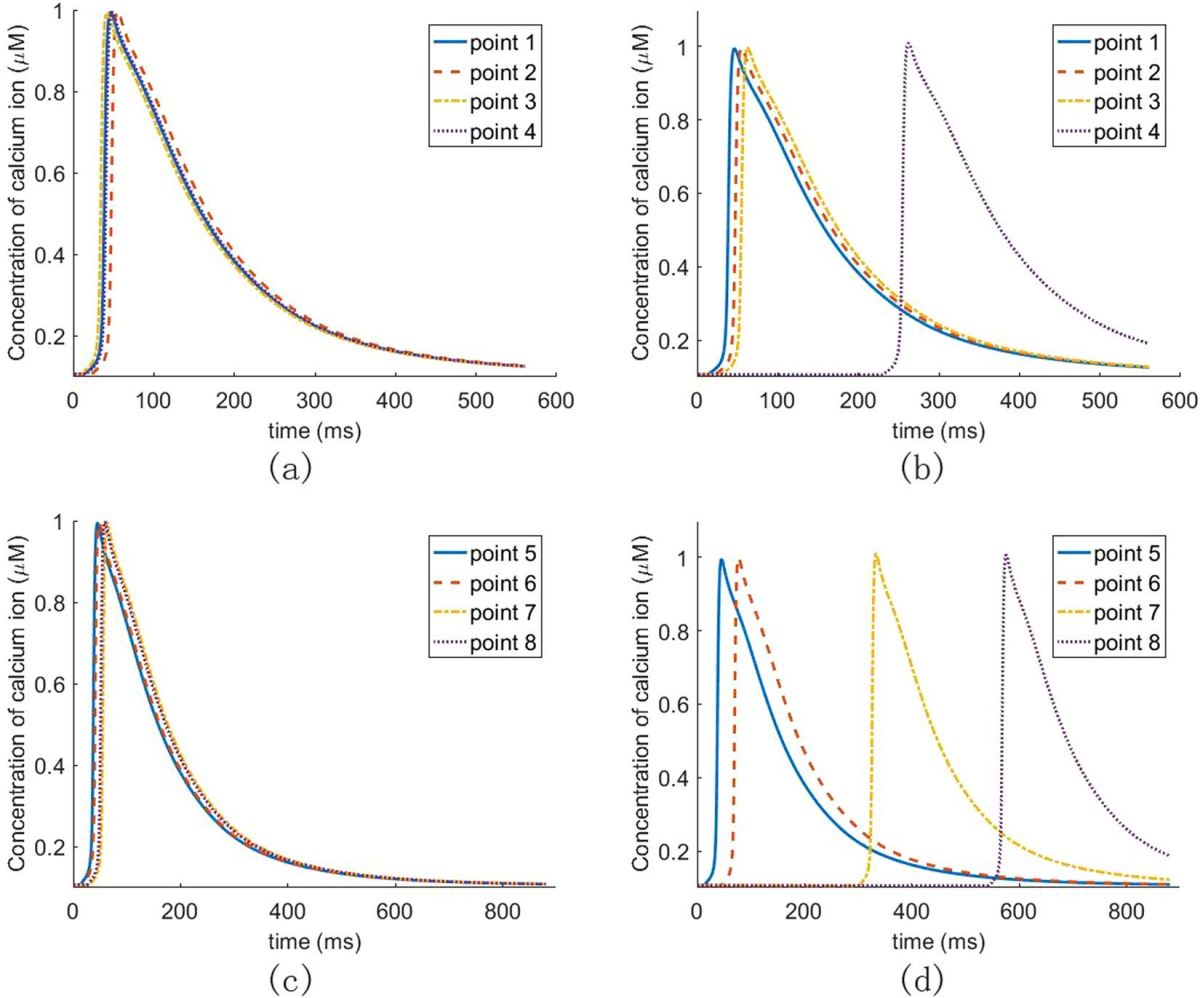
The concentration of calcium ion of human heart in different points: (**a**) four points in transverse section of the healthy heart; (**b**) four points in transverse section of the heart with LBBB; (**c**) four points in longitudinal section of the healthy heart; (**d**) four points in longitudinal section of the heart with LBBB.

To further explain how LBBB affect ventricular contraction, we select four points on the transverse and longitudinal sections, as shown in Fig. 6. Transmembrane potential and calcium ion concentration at each point are then analyzed. As shown in Fig. 9, the points in the healthy case are stimulated almost simultaneously, but not in the heart with LBBB, much delayed at points 4, 7 and 8. Comparing Fig. 9(a) with Fig. 9(b), the activation time of the point in the interventricular septum (such as point 2) in the LBBB case is similar to that in the healthy case. However, the activation time is significantly affected by LBBB at points away from the right ventricle. The farther away from the right ventricle, the later the activation. Figure 10 shows the calcium ion concentration at selected points. Since myofilament contraction is regulated by intracellular calcium transient, it can be seen from Fig. 10(a,c) that all points in the healthy cases can contract at the same time. While in the heart with LBBB (Fig. 10(a–d)), the points in the interventricular septum (e.g. points 2, 3 and 6) are essentially unaffected and will contract at nearly same time, but points 4 and 7 are about 250 *ms* late when they start to contract. For the point farthest from the right ventricle, namely point 8 at the lateral wall, the contraction time is about 600 *ms* later. Therefore, the LBBB will cause significant contraction delay in the left ventricular lateral wall, could potentially lead to heart failure in the long term.

## Discussion

The validity of the GS method is verified by some standard examples of the FitzHugh-Nagumo monodomain models. Based on the GS method, we capture the patterns of heterogeneity and complex connectivity of electrophysiological dynamics in biological tissues by solving the Fitzhugh-Nagumo monodomain model in rectangular and circular regions. The spiral wave obtained by the GS method is the same as that obtained by others, and it is verified that the GS method can effectively solve the monodomain model in the rectangular and circular regions. By comparing with the results in the stationary region, propagation velocity and shape of spiral waves in the moving region change. The propagation velocity in the moving region is higher than the velocity in the stationary region. The width of the spiral wave in the moving region is also slightly larger than the width in the stationary region. Since the GS method permits nonconforming discretization of the transmembrane potential and membrane dynamics, the monodomain model can be directly solved using finite different method in a regular ghost structure. Another advantage of the GS method is in dealing with the moving regions. Compared to Liu’s method^40^, the GS method needs to use the delta function to transform the Lagrangian and Eulerian variables, and the membrane dynamics need to be solved in a finer Lagrangian grid. In this sense, the GS method will require higher computational resource than Liu’s implementation^40^. For the stationary rectangular region, the computational time of the GS method is about 3 hours, since we have not fully optimized the implementation for high performance computing but only employed OpenMP functionality for dealing with “for”. It is expected that once we employ MPI and GPU computing, the computational time can be reduced significantly.

The AP propagation on the transverse and longitudinal sections of human heart is successfully simulated by the GS method. In a real heart, the AP transmits involves varieties of conduction cells, such as myocyte, sinoatrial node cells, atrioventricular nodal cells, Purkinje fibers, and fibroblasts. The electrical activity of the heart begins with the sinoatrial node at the right atrium. Pulses from the sinoatrial node travel through the left and right atrium and meet at the atrioventricular node. From the atrioventricular node, electrical impulses travel along the bundle and are transmitted to the right and left ventricles through the right and left bundle branches. Finally, the bundle at the end of the bundle branch is divided into millions of Purkinje fibers. Nowadays, many researchers have begun to study electrophysiology by including various conduction cells. The sinoatrial node is the normal pacemaker of the mammalian heart. There are a few mathematical models of sinoatrial nodes. For example, based on the Severi-DiFrancesco model of a rabbit sinoatrial node cell and the electrophysiological data from human sinoatrial node cells, Fabbri *et al*.^62^ proposed a comprehensive model of the electrical activity of a human sinoatrial node cell. The AP and CaT obtained by Fabbri were close to experimentally recorded values. In order to illustrate the functional role of various genetic isoforms of ion channels in generating cardiac pace-making AP, Kharche *et al*.^63^ developed a mathematical model for spontaneous AP of mouse sinoatrial node cells. In that model, biophysical properties of membrane ionic currents and intracellular mechanisms were considered. Results showed that their model could reproduce the physiological exceptionally short AP and high pacing rates of mouse sinoatrial node cells effectively.

Because of the importance of Purkinje system in both normal ventricular excitation and ventricular arrhythmias, modelling of the Purkinje system is essential for a realistic ventricle model of the heart^61^. Recently, inclusion of Purkinje network in AP modelling has attracted much attention^64^. For example, Oleg *et al*.^65^ developed a detailed model of the canine Purkinje-ventricular junction and varied its heterogeneity parameters to determine the relationship between wave conduction velocity, tissue structure, and safety of discontinuous conduction at nonuniform junctions. Oleg found that fast or slow conduction was unsafe, and there existed an optimal velocity that provided the maximum safety factor for conduction through the junction. Vergara *et al*.^66^ developed a model for the electrophysiology in the heart to handle the electrical propagation in the Purkinje system and myocardium. Their results illustrated the importance of using physiologically realistic Purkinje-trees for simulating cardiac activation. However, the majority of current anatomical models have not included models of the Purkinje network^61^. Instead, a simplified approach is adopted by applying electrical stimulus in the middle and lower third segments of the septum and endocardium^60^. The same approach is used in this study. This is partially due to the fact that extensive branching of the Purkinje fibers makes modelling Purkinje network extremely challenging as suggested by Tawara^59^, who carried out a formidable study lasting over 2 years to reconstruct the conduction system from experimental data. Since the focus of this study is to develop a numerical method for AP within myocardium, like many other studies^67,68^, we only consider myocytes, and other conduction cells are not simulated.

When the bundle branch is injured after myocardial infarction, or cardiac surgery, it may stop transmitting electrical impulses completely. This will result in a change in the path of ventricular depolarization. According to the anatomical location of the defect that leads to the bundle branch block, the block is further divided into the right bundle branch block and the left bundle branch block. Since the electrical pulse can no longer use the preferred path through the bundle branch, it can only spread through muscle fibers, which slows down the electrical propagation and changes the directional propagation of the electrical pulse. Lange *et al*.^44^ performed simulations to investigate the effect of different types of false tendons on hearts with the electrical conduction abnormality in a LBBB heart. Their results indicated that, LBBB affected the activation time of left and right ventricles, and the false tendon could compensate for the propagation delay caused by the LBBB. As demonstrated in this study, LBBB leads to delayed triggering of electrical excitation of the left ventricle, resulting in the loss of ventricular electrical synchrony, and potentially causing mechanical discoordination.

## Conclusion

In this study, we have developed a GS method by immerse the actual irregular electrophysiology computational domain into a larger rectangular region. Action potential propagation using the monodomain model is solved successfully with the GS method. In a rectangular and a circular regions, by using the GS method to solve the FitzHugh-Nagumo monodomain model, we capture the patterns of heterogeneity and complex connectivity of electrophysiological dynamics in biological tissues, and demonstrate the validity of the method. Numerical results show that the GS method can effectively simulate the AP propagation in irregular region. Furthermore, we employ the GS method to simulate the transmembrane propagation in moving regions and analyze the influence of moving region on transmembrane propagation. Our results show that the moving regions affect not only the propagation velocity but also the shape of spiral waves. Subsequently, we simulate the AP propagation on the transverse and longitudinal sections of a healthy heart and a heart with LBBB by using the GS method. The numerical results demonstrate how LBBB affects action prorogation in ventricles.

## Model Introduction

### Monodomain model

Simulating myocardial electrical activity needs to describe the anisotropic excitation conduction based on the ion channel model of myocardial cells. In general, a bidomain model based on the Poisson equation is used to describe the electrical coupling between myocytes and the electrical conduction cells in the tissue. At the microscopic level, myocardium can be seen as consisting of two separate regions separated by the cell membrane: the intracellular space (Ω_*i*_) and the extracellular space (Ω_*e*_). The bidomain model consists of the equations for the intracellular potentials (*ϕ*_*i*_) and the extracellular potentials (*ϕ*_*e*_), thus the transmembrane potential is *V*_*m*_ = *ϕ*_*i*_ − *ϕ*_*e*_. The governing equations of the bidomain model are

8 $$\nabla \cdot ({\sigma }_{i}\nabla {V}_{m})=-\,\nabla \cdot (({\sigma }_{i}+{\sigma }_{e})\nabla {\varphi }_{e})$$

9 $$\nabla \cdot ({\sigma }_{i}\nabla {V}_{m})+\nabla ({\sigma }_{i}\nabla {\varphi }_{e})={A}_{m}({C}_{m}\frac{\partial {V}_{m}}{\partial t}+{I}_{ion}+{I}_{s})$$

in which ${\sigma }_{i}={\rm{diag}}({\sigma }_{i}^{l},{\sigma }_{i}^{t})$ and ${\sigma }_{e}={\rm{diag}}({\sigma }_{e}^{l},{\sigma }_{e}^{t})$ are the conductivity tensor. *A*_*m*_ is the surface-to-volume ratio, i.e., the amount of membrane found in a given volume of tissue. *I*_*m*_ is the transmembrane current density. *C*_*m*_ is the membrane capacitance, *I*_*ion*_ is the ionic current through a number of different types of ion channels. *I*_*s*_ is an imposed stimulation current.

Assuming the anisotropy ratios in intracellular and extracellular spaces are the same, let *σ*_*e*_ = *λσ*_*i*_, then Eq. (8) will be reduced to

10 $$\nabla \cdot ({\sigma }_{i}\nabla {\varphi }_{e})=-\,\nabla \cdot (\frac{1}{1+\lambda }{\sigma }_{i}\nabla {V}_{m})$$

Substituting Eq. (10) into Eq. (9), we will obtain the governing equation of the monodomain model, that is

11 $$\frac{\partial {V}_{m}}{\partial t}=\frac{1}{{C}_{m}}(\frac{1}{{A}_{m}}(\frac{\lambda }{1+\lambda })\nabla \cdot ({\sigma }_{i}\nabla {V}_{m})-({I}_{ion}+{I}_{s}))$$

### Reaction-diffusion system

Both the bidomain model and the monodomain model are reaction-diffusion systems. They are singularly perturbed systems for model parameters and reasonable initial data^69^. When the diffusion phenomenon is included in the system, the threshold phenomenon ensures the stability of the traveling wave solution. The so-called threshold phenomenon, that is, there is a threshold value of the transmembrane potential *V*_*m*_ in the uniform space, for the electrophysiology model Eq. (9) or Eq. (11), when the potential is lower than the threshold value, it quickly returns to the stable state; when the potential is higher than the threshold value, it will produce a large excursion before it returns to the stable state. In cardiac tissue, an initial perturbation with a sufficiently large transmembrane potential *V*_*m*_ triggers AP propagation.

**Figure 11:**
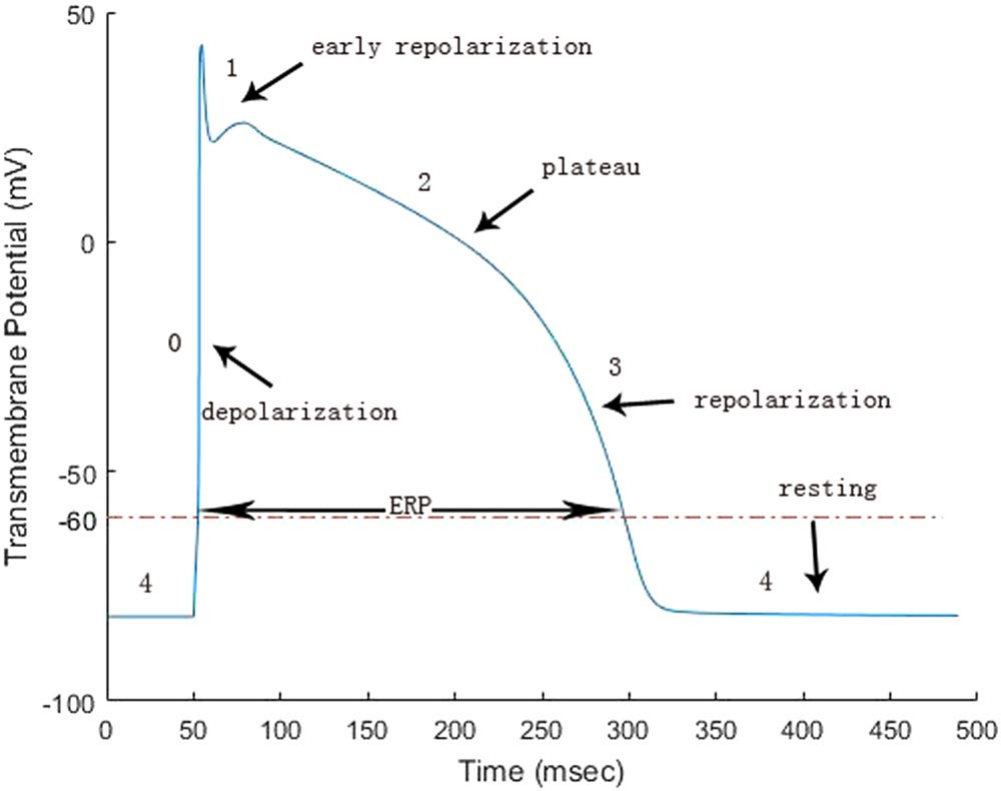
Sketch for the transmembrane potential *V*_*m*_.

Figure 11 shows a typical AP curve for a human myocyte, which consists of four phases, the depolarization phase (phase 0), the early repolarization phase (phase 1), the plateau phase (phase 2), the repolarization (phase 3), and the resting phase (phase 4). In a cardiac cycle, once a myocyte is excited, it can not be excited again for a period of time, the so-called effective refractory period (ERP). During this period, the depolarization of adjacent cardiomyocytes does not trigger already-excited myocytes. When entering the resting phase (phase 4), myocytes are ready for next excitation. ERP is usually characterized by the interval between the depolarization (phase 0) and repolarization (phase 3) phases. As a protective mechanism, ERP can control the heart rate, prevent arrhythmias and coordinate muscle contractions.

### The ghost structure method

**Figure 12:**
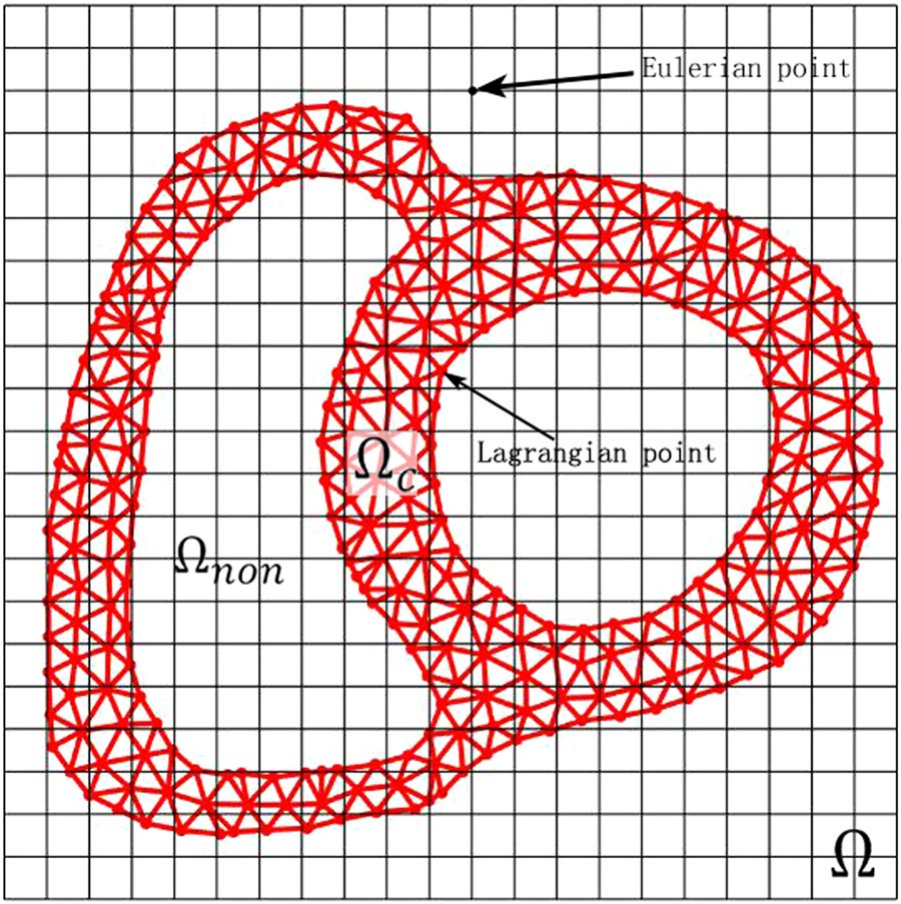
Sketch of the computational domain for the GS method.

In this ghost structure method, the transmembrane potential *V*_*m*_ is described by an Eulerian form and discretized with a regular Cartesian grid, while the membrane dynamics is described by a Lagrangian form and calculated by the cell membrane model. As shown in Fig. 12, the entire computational domain (the ghost structure region) is represented by Ω, where **X** = (**X**_1_, **X**_2_) ∈ **Ω**_*c*_ is Eulerian (physical) coordinates. The region where myocytes sit is expressed as Ω_*c*_, **X** = (**X**_1_, **X**_2_) ∈ Ω_*c*_ are fixed Lagrangian (material) coordinates. The mapping *χ*(**X**, *t*) ∈ Ω gives the physical position of each Lagrangian point at time *t*. Therefore, the physical region occupied by myocardium at time *t* is denoted as Ω_*c*_(*t*) = *χ*(**X**, *t*), and the region of non-myocardial cells at time *t* is denoted as Ω_*non*_(*t*) = Ω − Ω_*c*_. The Lagrangian and Eulerian variables are transformed by the integral transformation of the delta function. Finally, the full governing equations of the monodomain model in the GS method are

12 $$\frac{\partial {V}_{m}({\bf{x}},t)}{\partial t}=\frac{1}{{C}_{m}}\{\frac{1}{{A}_{m}}(\frac{\lambda }{1+\lambda })\nabla \cdot [{\sigma }_{i}\nabla {V}_{m}({\bf{x}},t)]-[{I}_{ion}({\bf{x}},t)+{I}_{s}({\bf{x}},t)]\}$$

13 $${\tilde{V}}_{m}({\bf{X}},t)={\int }_{{\rm{\Omega }}}\,{V}_{m}({\bf{x}},t)\delta ({\bf{x}}-\chi ({\bf{X}},t))d{\bf{x}},$$

14 $$\frac{\partial y({\bf{X}},t)}{\partial t}=f({\tilde{V}}_{m}({\bf{X}},t),y({\bf{X}},t))$$

15 $${\tilde{I}}_{ion}({\bf{X}},t)=g({\tilde{V}}_{m}({\bf{X}},t),y({\bf{X}},t))$$

16 $${I}_{ion}({\bf{x}},t)={\int }_{{{\rm{\Omega }}}_{c}}\,{\tilde{I}}_{ion}({\bf{X}},t)\delta ({\bf{x}}-\chi ({\bf{X}},t))d{\bf{X}},$$

17 $${I}_{s}({\bf{x}},t)={\int }_{{{\rm{\Omega }}}_{c}}\,{\tilde{I}}_{s}({\bf{X}},t)\delta ({\bf{x}}-\chi ({\bf{X}},t))d{\bf{X}},$$

where *V*_*m*_(**x**, *t*) is the Eulerian transmembrane potential, *I*_*ion*_(**x**, *t*) is the Eulerian ionic current, and *I*_*s*_(**x**, *t*) is the Eulerian stimulation current density. ${\tilde{V}}_{m}({\bf{X}},t)$, ${\tilde{I}}_{ion}({\bf{X}},t)$, and ${\tilde{I}}_{s}({\bf{X}},t)$ are the transmembrane potential, ionic current and stimulation current density in Lagrangian form, respectively. *y* is a Lagrangian vector of ionic fluxes and their corresponding channel gating variables are described by the suitable ordinary differential equations Eq. (14). **f** represents the right hand side of the ordinary differential equations used to describe ion channels. *g* represents a nonlinear function that relates the ionic flux to the total ionic current. Eq. (14) and Eq. (15) are cardiac membrane models used to solve the current-voltage relationship. *δ*(**x**) is a two-dimensional delta function that transforms the transmembrane potential and ion current between the Eulerian and Lagrangian coordinates. In this study, we calculate ion currents by Eq. (13) and Eq. (14) through the GPB model, and then convert ${\tilde{I}}_{ion}({\bf{X}},t)$ into the Eulerian ion current in the ghost structure region. Finally, the Eulerian transmembrane potential *V*_*m*_(**x**, *t*) is obtained by solving Eq. (12).

### Spatial discretization

**Figure 13:**
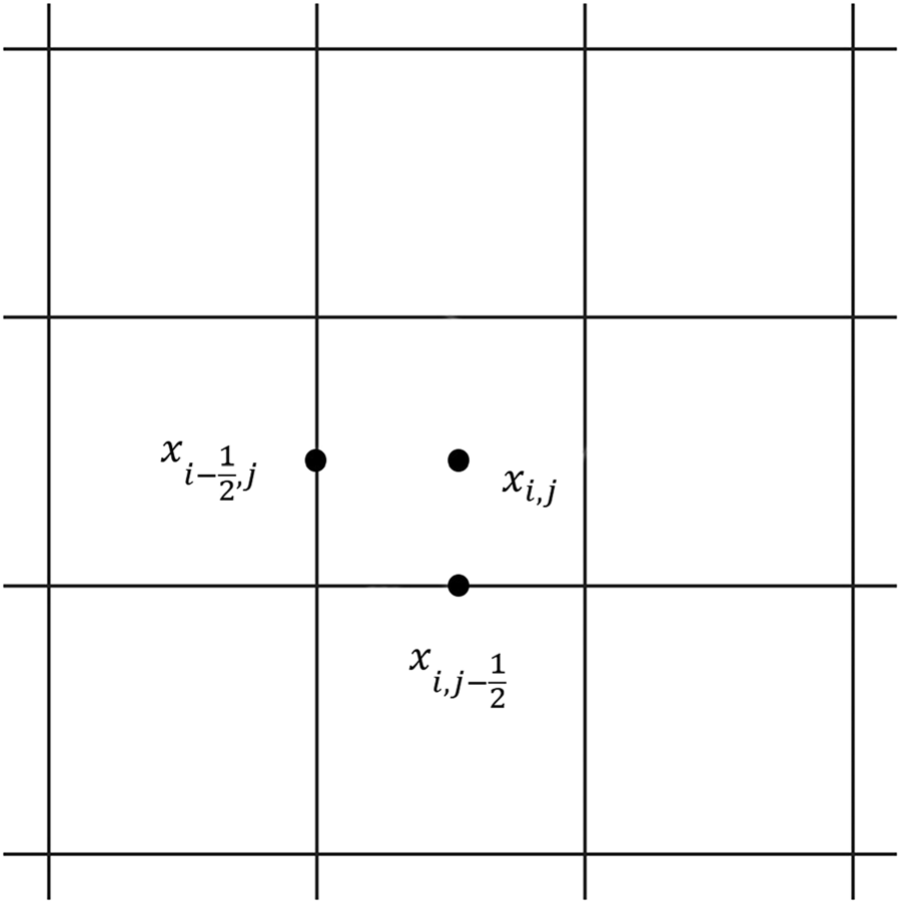
Sketch of the staggered-grid.

The regular ghost structure region Ω is discrete by a *N*_1_ × *N*_2_ Cartesian grid with spatial steps ${\rm{\Delta }}{x}_{1}=\frac{1}{{N}_{1}}$ and ${\rm{\Delta }}{x}_{2}=\frac{1}{{N}_{2}}$. The center of the Cartesian grid is represented as ${{\bf{x}}}_{i,j}=((i+\frac{1}{2}){\rm{\Delta }}{{\bf{x}}}_{1},(j+\frac{1}{2}){\rm{\Delta }}{x}_{2})$, where *i* = 0, ..., *N*_1_−1, *j* = 0, ..., *N*_2_−1. The transmembrane potential *V*_*m*_ and the current *I*_*ion*_ are defined at the center of the grid and are represented by (*V*_*m*_)_*i*,*j*_ and (*I*_*ion*_)_*i*,*j*_, respectively. The divergence of ∇*V*_*m*_ is approximated at the central point of the grid, as illustrated in Fig. 13, ${{\bf{x}}}_{i-\frac{1}{2},j}=(i{\rm{\Delta }}{x}_{1},(j-\frac{1}{2}){\rm{\Delta }}{x}_{2})$ and ${x}_{i,j-\frac{1}{2}}=((i-\frac{1}{2})\Delta {x}_{1},j\Delta {x}_{2})$.

The gradient of *V*_*m*_ is approximated at the center point of the grid again using the following difference scheme,

$$\nabla {V}_{m}=({\nabla }_{{x}_{1}}{V}_{m},{\nabla }_{{x}_{2}}{V}_{m})$$

$${({\nabla }_{{x}_{1}}{V}_{m})}_{i-\frac{1}{2},j}=\frac{{({V}_{m})}_{i,j}-{({V}_{m})}_{i-\mathrm{1,}j}}{{\rm{\Delta }}{x}_{1}}$$

$${({\nabla }_{{x}_{2}}{V}_{m})}_{i,j-\frac{1}{2}}=\frac{{({V}_{m})}_{i,j}-{({V}_{m})}_{i,j-1}}{{\rm{\Delta }}{x}_{2}}$$

The divergence of *σ*_*i*_∇*V*_*m*_ is defined also at the centre of the grid as

$$\nabla \cdot ({\sigma }_{i}\nabla {V}_{m})={\nabla }_{{x}_{1}}({\sigma }_{i}^{l}{\nabla }_{{x}_{1}}{V}_{m})+{\nabla }_{{x}_{2}}({\sigma }_{i}^{t}{\nabla }_{{x}_{2}}{V}_{m})$$

$$\begin{array}{rcl}{({\nabla }_{{x}_{1}}({\sigma }_{i}^{l}{\nabla }_{{x}_{1}}{V}_{m}))}_{i,j} & = & \frac{{({\sigma }_{i}^{l}{\nabla }_{{x}_{1}}{V}_{m})}_{i+\frac{1}{2},j}-{({\sigma }_{i}^{l}{\nabla }_{{x}_{1}}{V}_{m})}_{i-\frac{1}{2},j}}{{\rm{\Delta }}{x}_{1}}\\  & = & {\sigma }_{i}^{l}\frac{{({V}_{m})}_{i+\mathrm{1,}j}-\mathrm{2(}{V}_{m}{)}_{i,j}+{({V}_{m})}_{i-\mathrm{1,}j}}{{\rm{\Delta }}{{x}_{1}}^{2}}\\ {({\nabla }_{{x}_{2}}({\sigma }_{i}^{t}{\nabla }_{{x}_{2}}{V}_{m}))}_{i,j} & = & \frac{{({\sigma }_{i}^{t}{\nabla }_{{x}_{2}}{V}_{m})}_{i,j+\frac{1}{2}}-{({\sigma }_{i}^{t}{\nabla }_{{x}_{2}}{V}_{m})}_{i,j-\frac{1}{2}}}{{\rm{\Delta }}{x}_{2}}\\  & = & {\sigma }_{i}^{t}\frac{{({V}_{m})}_{i,j+1}-\mathrm{2(}{V}_{m}{)}_{i,j}+{({V}_{m})}_{i,j-1}}{{\rm{\Delta }}{{x}_{2}}^{2}}\end{array}$$

The initial value of *V*_*m*_ at each Eulerian point in the entire computational domain needs to be approximated based on the transmembrane potential at the Lagrangian point and the integral transformation of the delta function. In order to solve more accurately, the transmembrane potential at the Lagrangian point in Ω_*c*_ and the delta function are required to update the transmembrane potential at the Eulerian point outside the structure Ω_*c*_ at regular intervals. The mutual transformation between the Eulerian variable and the Lagrangian variable will be explained in the subsection “Lagrangian-Eulerian interaction”.

### Time discretization

For the ordinary differential equations Eq. (14), the third-order TVD Runge-Kutta method^70^ is used to solve the system:

18 $${{\bf{y}}}^{\mathrm{(1)}}={{\bf{y}}}^{n}+{\rm{\Delta }}t\,f({{\tilde{V}}_{m}}^{n+1},{{\bf{y}}}^{n})$$

19 $${{\bf{y}}}^{\mathrm{(2)}}={{\bf{y}}}^{\mathrm{(1)}}+\frac{{\rm{\Delta }}t}{4}[-3f({{\tilde{V}}_{m}}^{n+1},{{\bf{y}}}^{n})+f({{\tilde{V}}_{m}}^{n+1},{{\bf{y}}}^{\mathrm{(1)}})]$$

20 $${{\bf{y}}}^{n+1}={{\bf{y}}}^{\mathrm{(2)}}+\frac{{\rm{\Delta }}t}{12}[-f({{\tilde{V}}_{m}}^{n+1},{{\bf{y}}}^{n})-f({{\tilde{V}}_{m}}^{n+1},{{\bf{y}}}^{\mathrm{(1)}})+8f({{\tilde{V}}_{m}}^{n+1},{{\bf{y}}}^{\mathrm{(2)}})]$$

For the nonlinear partial differential equation Eq. (12), it can be rewritten as

21 $$\frac{\partial {V}_{m}}{\partial t}=L({V}_{m})$$

where, $L({V}_{m})=\frac{1}{{C}_{m}}[\frac{1}{{A}_{m}}(\frac{\lambda }{1+\lambda })\nabla \cdot ({\sigma }_{i}\nabla {V}_{m})-({I}_{ion}+{I}_{s})]$. In this paper, the third-order TVD Runge-Kutta method is also used to solve the nonlinear reaction-diffusion equation Eq. (21)

22 $${V}_{m}^{\mathrm{(1)}}={V}_{m}^{n}+{\rm{\Delta }}tL({V}_{m}^{n+1})$$

23 $${V}_{m}^{\mathrm{(2)}}={V}_{m}^{\mathrm{(1)}}+\frac{{\rm{\Delta }}t}{4}[-\,3L({V}_{m}^{n})+L({V}_{m}^{\mathrm{(1)}})]$$

24 $${V}_{m}^{n+1}={V}_{m}^{\mathrm{(2)}}+\frac{{\rm{\Delta }}t}{12}[-\,L({V}_{m}^{n})-L({V}_{m}^{\mathrm{(1)}})+8L({V}_{m}^{\mathrm{(2)}})]$$

### Lagrangian-Eulerian interaction

Let *τ*_*h*_ = ∪Ω^*e*^ be a triangulation of Ω_*c*_, in which *e* indexes the elements of the mesh of the irregular computational domain, The nodes of mesh are denoted as ${\{{{\bf{X}}}_{l}\}}_{l\mathrm{=1}}^{M}$, the finite element basis functions are denoted by ${\{{\varphi }_{l}({\bf{X}})\}}_{l\mathrm{=1}}^{M}$. In this paper, nodes of the mesh are regarded as Lagrangian points. The Lagrangian points must be finer than the Cartesian points to avoid leaks^71^. In the calculation process, the integral transformation form of delta function is used to realize the conversion between the Eulerian variable and Lagrangian variable. The approximation of the smooth delta function is ${\delta }_{h}({\bf{x}})={\prod }_{i\mathrm{=1}}^{d}\,{{\rm{\Psi }}}_{h}({r}_{i})$, ${r}_{i}=\frac{{x}_{i}}{{\rm{\Delta }}{x}_{i}}$, which is a regularization function of a four-point smooth delta function proposed by Gong^72^,

25 $${{\rm{\Psi }}}_{h}({r}_{i})=\{\begin{array}{ll}\frac{1}{2}(|{r}_{i}|+\mathrm{1)(}|{r}_{i}|-\mathrm{1)(}|{r}_{i}|-\mathrm{2),} & |{r}_{i}|\le 1\\ -\,\frac{1}{6}(|{r}_{i}|-\mathrm{1)(}|{r}_{i}|-\mathrm{2)(}|{r}_{i}|-\mathrm{3),} & 1\le |{r}_{i}|\le 2\\ \mathrm{0,} & other\end{array}$$

Based on the above delta function, the approximate values of physical quantities at each Eulerian point can be obtained directly by using the values of Lagrangian points around the Eulerian point. In order to obtain the approximate value of physical quantity in Eulerian coordinate system more accurately, we use the Gaussian quadrature rule with quadrature points ${{\bf{X}}}_{Q}^{e}$, where ${{\bf{X}}}_{Q}^{e}\in {{\rm{\Omega }}}^{e}$, and accociated weights ${\omega }_{Q}^{e}(Q=\mathrm{1,}\,\mathrm{...,}\,{N}^{e})$. In the GS method, the value of each Gaussian integral point is obtained through the basis function of the finite element mesh, and then the approximate value of the physical quantity at the Eulerian point is obtained by using the integral transformation form of the delta function. Taking *I*_*ion*_(**x**, *t*) as an example, the current ${\tilde{I}}_{ion}$ of the Gaussian integration point in the element is obtained by the value of the Lagrangian point through the element basis function of the finite element method, and the approximate value *I*_*ion*_ of the ${\tilde{I}}_{ion}$ at the Eulerian point is

26 $${I}_{ion}({\bf{x}},t)=\sum _{{{\rm{\Omega }}}^{e}\in {\tau }_{h}}\,\sum _{Q=1}^{{N}^{e}}\,{\tilde{I}}_{ion}({{\bf{X}}}_{Q}^{e})\delta ({\bf{x}}-\chi ({{\bf{X}}}_{Q}^{e},t)){\omega }_{Q}^{e}$$

Similarly, we can use the integral transformation form of the corresponding delta function to obtain the approximate value of *V*_*m*_ at each Gaussian integral point by using the transmembrane potential *V*_*m*_ at Eulerian points, that is

27 $${\tilde{V}}_{m}({{\bf{X}}}_{Q}^{e},t)=\sum _{i,j}\,{V}_{m}({{\bf{x}}}_{i,j})\delta ({{\bf{x}}}_{i,j}-\chi ({{\bf{X}}}_{Q}^{e},t)){\rm{\Delta }}{x}_{1}{\rm{\Delta }}{x}_{2}$$

In order to update the ion current at the next time step through the cell membrane model, we need to obtain the transmembrane potential ${\tilde{V}}_{m}$ at the Lagrangian point or the grid nodes. The transmembrane potential ${\tilde{V}}_{m}$ at grid nodes is obtained by using the approximate value of Gaussian integral point from Eq. 27 and a *L*^2^ projection method^48^.

All simulations are performed on a windows workstation with Intel(R) Xeon(R) Gold 5115 (20 cores, 2.40 GHz, 64 GB memory), implemented in C++.
